# Electrolyte-Supported Fuel Cell: Co-Sintering Effects of Layer Deposition on Biaxial Strength

**DOI:** 10.3390/ma12020306

**Published:** 2019-01-18

**Authors:** Alessia Masini, Thomas Strohbach, Filip Šiška, Zdeněk Chlup, Ivo Dlouhý

**Affiliations:** 1 Institute of Physics of Materials, Academy of Science of the Czech Republic, 61662 Brno, Czech Republic; siska@ipm.cz (F.Š.); chlup@ipm.cz (Z.C.); idlouhy@ipm.cz (I.D.); 2 Sunfire GmbH, 01237 Dresden, Germany; Thomas.Strohbach@sunfire.de

**Keywords:** SOC, mechanical strength, flexural biaxial test, ball-on-3-balls test, fractography, residual stresses

## Abstract

The mechanical reliability of reversible solid oxide cell (SOC) components is critical for the development of highly efficient, durable, and commercially competitive devices. In particular, the mechanical integrity of the ceramic cell, also known as membrane electrolyte assembly (MEA), is fundamental as its failure would be detrimental to the performance of the whole SOC stack. In the present work, the mechanical robustness of an electrolyte-supported cell was determined via ball-on-3-balls flexural strength measurements. The main focus was to investigate the effect of the manufacturing process (i.e., layer by layer deposition and their co-sintering) on the final strength. To allow this investigation, the electrode layers were screen-printed one by one on the electrolyte support and thus sintered. Strength tests were performed after every layer deposition and the non-symmetrical layout was taken into account during mechanical testing. Obtained experimental data were evaluated with the help of Weibull statistical analysis. A loss of mechanical strength after every layer deposition was usually detected, with the final strength of the cell being significantly smaller than the initial strength of the uncoated electrolyte (*σ*_0_ ≈ 800 MPa and *σ*_0_ ≈ 1800 MPa, respectively). Fractographic analyses helped to reveal the fracture behavior changes when individual layers were deposited. It was found that the reasons behind the weakening effect can be ascribed to the presence and redistribution of residual stresses, changes in the crack initiation site, porosity of layers, and pre-crack formation in the electrode layers.

## 1. Introduction

Reversible solid oxide cells (SOCs) are devices able to produce synthetic fuels when operated in electrolysis mode (SOEC) and electricity when operated in fuel cell mode (SOFC). Recently, SOCs have been attracting a lot of interest because of their environmentally-friendly nature, their flexibility with respect to the fuel utilization and energy source integration, their capability and their surprisingly high overall efficiency [1,2,3,4]. They represent a promising tool towards a sustainable future.

A major threat hindering the successful commercialization of SOCs is their long-term reliability. Because of their high operating temperature (about 850 °C), the harsh oxidizing and reducing working atmospheres, while being subjected to external mechanical loads, their integrity is threatened [5,6]. 

A SOC device consists of ceramic, metallic, and glass components all stacked together. The mechanical failure of the ceramic cell, also known as MEA (membrane electrolyte assembly), would be detrimental for the proper functioning of the whole device. Being the component in which all the electrochemical reactions take place, its failure would inevitably lead to decreased performance of the entire stack. In order to ensure the efficiency of the SOC device, it is fundamental that the fuel and the oxidizing air are physically separated; their separation is ensured by the gastight electrolyte. This way, the fuel is not directly burned off. Any kind of leakage would reduce the quantity of effectively utilized fuel and therefore lower the efficiency. If the electrolyte breaks, the necessary gas tightness is no longer maintained and the SOC performance is hindered [7]. This exemplifies the importance of ensuring the mechanical integrity of the cell and in particular of the electrolyte over the whole expected lifetime. 

This work deals with the effect of the manufacturing process on the final strength of the ceramic cell. The design of this multi-layered ceramic system is mainly focused on the electrochemical properties necessary to make it an effective means for the production of electricity and synthetic fuels. However, it also requires certain robustness to be able to bear the severe mechanical and thermal stresses to which it is exposed during service [8]. The cell is a ceramic component mainly consisting of a dense electrolyte, which in this study is the supportive layer, embedded between two porous electrodes. During the production process, these functional layers are sintered together [9]. Because of the mismatch in the coefficient of thermal expansion, residual stresses will arise between the layers. Such stresses might be responsible for the formation of cracks in the porous electrodes, as well as for layer delamination, potentially compromising the overall strength of the ceramic cell [8,10,11]. The overall robustness of the cell under investigation, being of the electrolyte-support kind, depends on the properties of the electrolyte in the first place. Yet, its strength is also influenced by the features of the electrodes embedding the electrolyte and by the interfaces generated between them. 

Despite most of the research activity for the development of fuel cells being devoted to the electrochemical aspects, there are some studies dealing with the fracture mechanics of materials involved in SOC technology. However, nearly all of them are focused on the characterization of individual materials [12,13,14,15,16] or they investigate either symmetrical systems or half-cell systems [7,10,17,18,19,20]. As already mentioned, interfacial bonding between layers can play a significant role in the mechanical response of the whole cell. Thus, it is of high importance to understand the fracture mechanism of such a fundamental SOC component in its totality, treating it as a whole system, thus taking into account co-sintering effects and interaction between layers. This challenging approach is the main novelty of the presented work. 

It has already been reported that the strength of the electrolyte on the cell level is sensibly reduced [7,10]. The goal of this work is to understand the reasons behind this strength loss, aiming toward the improvement of the cell mechanical stability.

## 2. Materials and Methods

### 2.1. Specimen Preparation 

**Figure 1:**
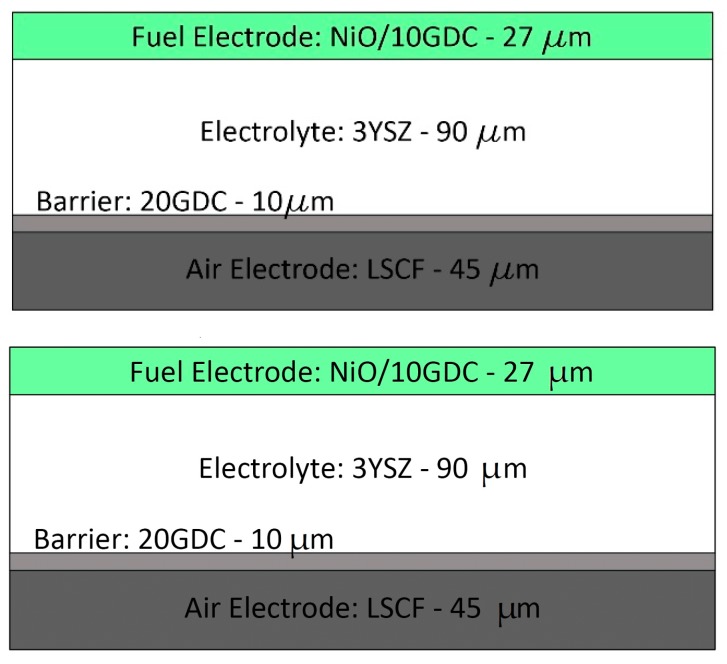
Schematic representation of the Sunfire MEA with its functional layers.

The planar electrolyte-supported SOC cell investigated in this study was provided by Sunfire (Sunfire GmbH, Dresden, Germany) and consisted of four layers; its layout is schematically represented in Figure 1. The detailed composition of each layer, together with the nominal thickness, is reported in Table 1. The planar electrolyte was produced via tape casting [21] and provided by the company Kerafol (KERAFOL Keramische Folien GmbH, Eschenbach in der Oberpfalz, Germany); it consisted of dense 3 mol% Y_2_O_3_-stabilized ZrO_2_ with a nominal thickness of 90 µm and it provided the mechanical support for the electrodes. Both the electrodes were manufactured at Sunfire (Sunfire GmbH, Dresden, Germany) via a screen printing process. The fuel electrode was a 27 µm thick porous NiO/Gd_0.1_Ce_0.9_O_2_ cermet, while the air electrode consisted of two layers: a 10-µm thick Gd_0.2_Ce_0.8_O_2_ barrier layer [22] and a La_0.6_Sr_0.4_Co_0.2_Fe_0.8_O_3−δ_ functional layer with a nominal thickness of 45 µm.

All the layers added to the electrolyte are sintered together. Thus, the cell is a complex system made up of co-sintered individual layers and its overall properties are inevitably affected by the constraints arising between them. To investigate the effect of these constraints on the mechanical response of the cell, samples were taken out from each production step (i.e., after each layer deposition in the green state) prior to sintering. Then laminates having two, three, and four layers were sintered via the same profile used for the whole cell. This approach led to the production of three non-symmetric layered structures, each of them with a different number of layers, and enabled the detection of the interactions between them. The layered structures were named from SOC1, corresponding to the electrolyte with barrier layer, to SOC3, corresponding to the whole cell, as illustrated in Table 2. SOC0 refers to the monolithic bare 3YSZ (3% mol Ytttria Stabilized Zirconia) electrolyte support. Mechanical characterizations were performed after each layer deposition, (i.e., on each layered structure from SOC0 to SOC3); in order to take into account the non-symmetric and non-periodic layer placement, both laminate sides were subject to testing (i.e., eight configurations were evaluated in total).

For the biaxial flexural testing, samples were extracted directly from SOC0–SOC3 as-sintered plates of dimensions 100 × 150 mm^2^ according to Table 2. The plates, being extremely thin and fragile, were glued onto a rigid support and cut into 4 x 3 mm^2^ rectangular specimens using a precision diamond saw Isomet 5000 (Buehler, Lake Bluff, IL, USA). The cutting speed was set to 7.8 mm/min in order to prevent the cracking of edges. The edges of the samples did not need any further polishing as, during the biaxial flexural test used, samples were subjected to tensile stresses concentrated in the central area located in between the loading balls; thus, any micro-cracks in correspondence of edges had no influence on the fracture load.

### 2.2. Biaxial Flexural Strength Test

**Figure 2:**
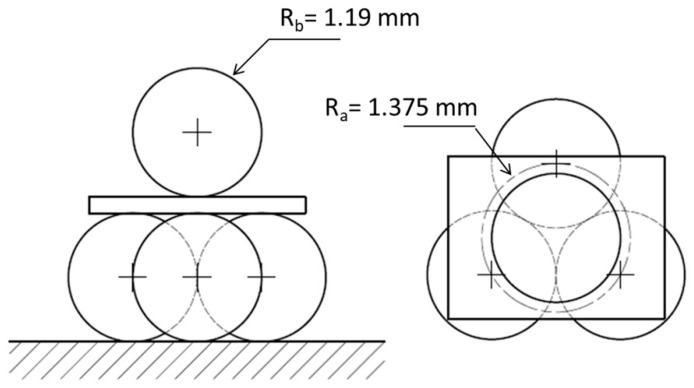
Scheme of the ball-on-3-balls test setup for biaxial testing.

**Figure 3:**
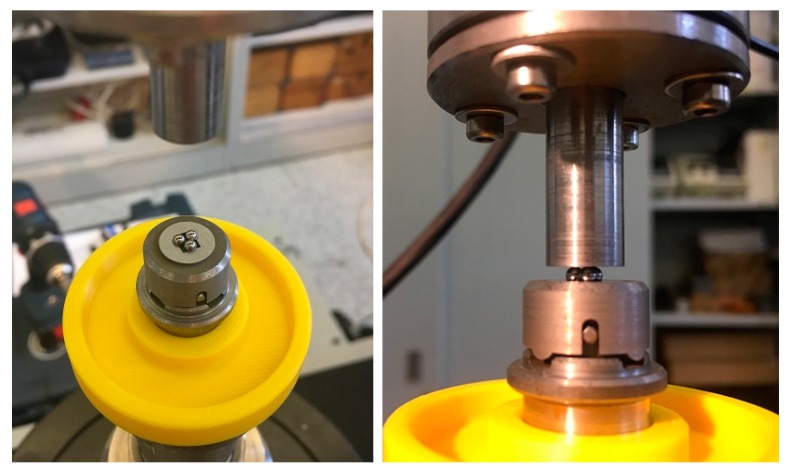
The test setup used for the measurements of the bi-axial flexural strength via B3B.

The biaxial flexural strength was determined through the ball-on-3-balls bending (B3B) configuration. Details of the testing procedure and setup can be found elsewhere [7,23,24]. The measurements were performed at room temperature in air atmosphere on rectangular specimens, which were symmetrically supported by three balls on one side and loaded by a fourth ball placed in the center of the opposite side (see Figure 2); all the balls were made of hardened steel (E_b_ = 210 GPa; ν_b_ = 0.3) and had a diameter R_b_ = 2.38 mm, giving a support radius R_a_ = 1.3747 mm. All B3B tests were performed under displacement control in a universal testing machine INSTRON 8862 (Instron, Norwood, MA, USA) with the aid of a jig especially produced at IPM (Institute of Physics of Materials, Brno, Czech republic), following the design from ISFK (Institut of Structural and Functional Ceramics, Leoben, Austria) [11]. During the test, the required alignment between the specimen, loading ball, and supporting balls was ensured by a guide, which was carefully removed after pre-load. The load was then further increased until failure [25]. The crosshead speed of the test was set to 500 µm/min in order to achieve the fracture of the samples in less than 5 s. The test setup utilized is shown in Figure 3. The minimum of 45 valid tests was conducted. Each configuration (SOC0–SOC3) was tested on both sides to take into account two aspects:

1. The electrolyte had a different surface refinement at the top and bottom side due to the manufacturing process; one side was smoother and the other is rougher, depending on whether it was on the support or the doctor blade side. This aspect may have led to a difference in the strength between two sides even when the electrolyte was a dense monolithic ceramic;
2. The layered structures SOC1 to SOC3 had a non-symmetrical non-periodic layout.

Experimental data were evaluated according to the Weibull statistical analysis. This was to take into account the inherently scattered nature of the strength of brittle materials, which cannot be described by a single strength value, but by a strength distribution. The characteristic strength *σ*_0_ and the Weibull modulus *m* of the Weibull distribution, together with their 95%-confidence intervals, were determined using the maximum-likelihood method, following the standard EN 843-5 [26]. Calculations were performed with the help of the statistical software Statgraphics Centurion 18 (Statgraphics Technologies, Inc., The Plains, VA, USA). 

After the tests, fractographic analyses were performed for every data set in order to characterize the fracture mechanisms acting and to investigate the effect of the layered layout on the crack propagation. The fracture surfaces of specimens exhibiting the highest and lowest values within the dataset were observed. For the fractographic analyses, fractured specimens were mounted in the specially prepared holder via silver paste and coated with a thin carbon film in order to give them the required conductivity for enabling scanning electron microscopy (SEM) observations. A scanning electron microscope Tescan LYRA 3 XMU (Tescan Brno, s.r.o., Brno, Czech Republic) was used. All the observations were performed at a working distance of 9 mm with an acceleration voltage of 20 kV.

### 2.3. Determination of the Flexural Strength

**Figure 4:**
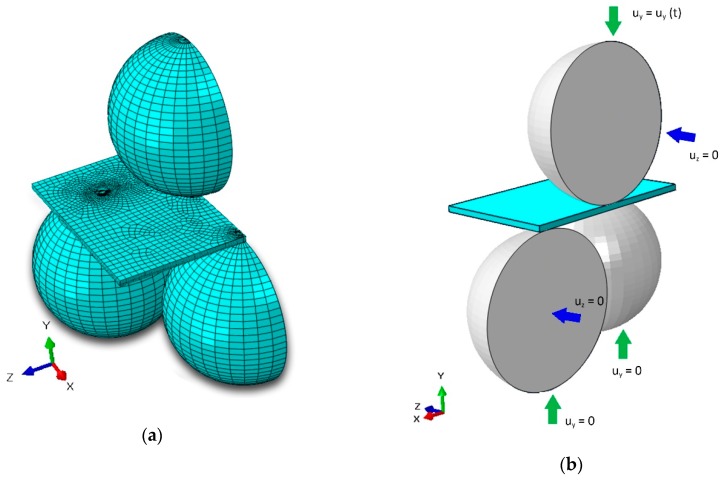
Finite Element (FE)-model example of the ball on three balls test assembly, half model: (**a**) view of the meshed model, and (**b**) outlined boundary conditions.

The flexural strength (in N/mm^2^) was determined from the experimental fracture force measured for each sample, via the equation:

(1) $$\sigma_{max}=f\cdot \frac{F}{t^{2}}$$

where *F* (N) is the maximum load at fracture; *t* (mm) the thickness of the specimen; and *f* is a dimensionless factor depending on the geometry of the specimen, its Poisson’s ratio, and the geometry of the test jig. Considering that the thickness is one of the most influential parameters for the estimation of the maximum stress, it was carefully measured in the center of all specimens (i.e., area where the maximum stress is located) before testing. To determine the *f* factor for each tested material configuration loaded using B3B, an FEM (Finite Elements Method) analysis was performed using the commercial software Abaqus/CAE6.13 (Dassault Systemes Simulia Corp., Providence, RI, USA). For the simulation, the rectangular samples and the balls were modelled using 3D deformable elements of the C3D8R type. Given the symmetry of the system, only half of the testing setup was modelled in order to save computational time. The chosen geometry and boundary conditions are illustrated in Figure 4. The mesh in the model was created in order to combine sufficient precision and reasonable computational demands. Therefore, the areas of contact between the balls and the cell were meshed more densely with the in-plane element size from 2 µm to 10 µm. The rest of the cell was meshed with increasing element size (up to 100 µm). The average through thickness element size was 4 µm; however, there were at least two elements through the thickness of the layer. The number of DOF (Degree of Freedom) for the cell ranged between 252 000 (SOC0) and 468 000 (SOC3). Siska et al. [27] showed that for elastic calculations of heterogeneous material, the mesh convergence is achieved at around 100 000 DOF. Therefore, the performed simulations were well conditioned in the sense of mesh convergence.

Material data used for the simulations are reported in Table 3. Elastic modulus E, Poisson’s ratios ν, and densities ρ were taken from Reference [4], while coefficients of thermal expansion α were measured via dilatometry or taken from literature [28,29]. 

**Figure 5:**
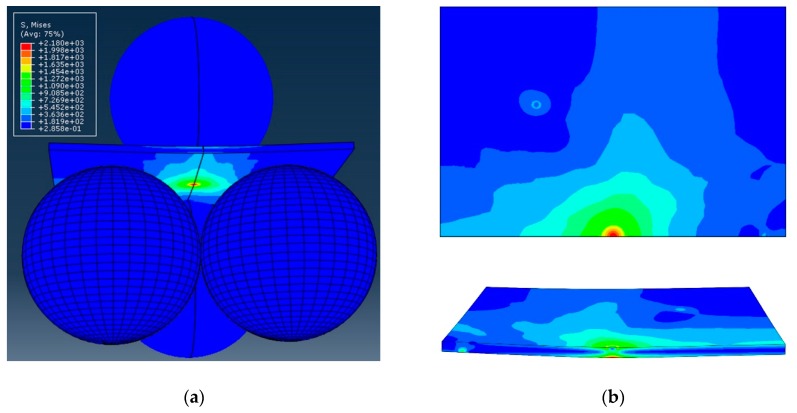
Example of the principal stress field in a rectangular plate specimen for a typical loading condition in a ball-on-3-balls test: (**a**) perspective view, and (**b**) view on the top plane and section view of the plate.

In Figure 5, an example of the first maximum principal stress distribution in the specimen during biaxial loading is represented. It can be observed that the maximum stress arose in the center of the tensile surface of the specimen (the red area), corresponding to the center of the three balls, and its intensity decreased sharply in the radial direction. Therefore, as the area loaded with the maximum tensile stress was a small portion of the volume of the sample, localized strength measurements could be carried out.

## 3. Results

### 3.1. Flexural Strength Measurement

**Figure 6:**
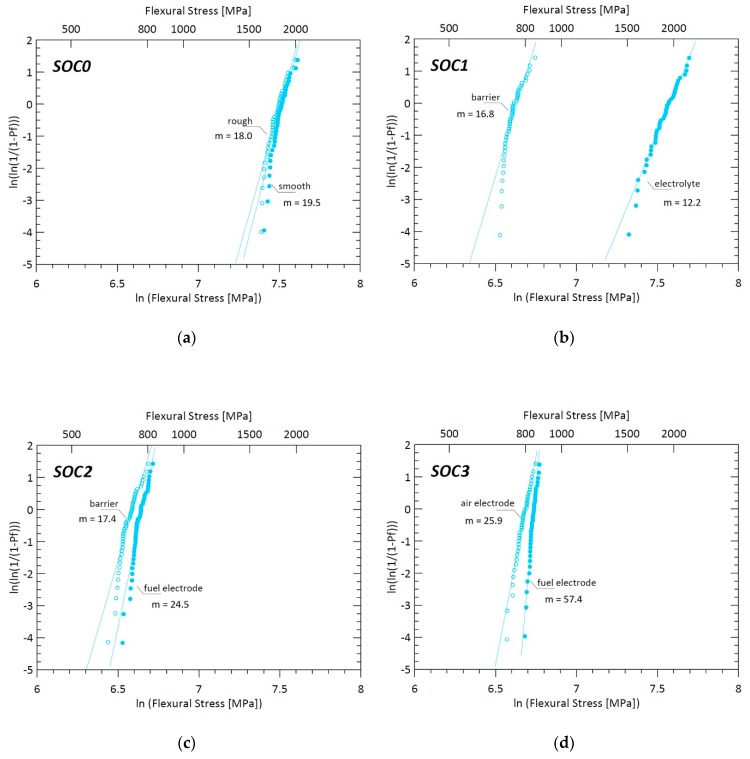
Weibull plots of the fracture stress distribution of the SOC0-SOC3 samples, obtained via ball-on-3-balls test. In each graph are represented two plots, one for each side of the sample under tension, with their respective Weibull modules. (**a**): Weibull plot for SOC0 (**b**): Weibull plot for SOC1 (**c**): Weibull plot for SOC2 (**d**): Weibull plot for SOC3.

The failure stresses of the samples in the flexural B3B configuration are illustrated in Figure 6 in the form of Weibull plots and summarized in Table 3, where characteristic strengths and Weibull modules are reported together with their calculated confidence intervals. For the electrolyte (SOC0), the characteristic strength ranged between *σ*_0_ = 1819 MPa (rough side under tension) and *σ*_0_ = 1854 MPa (smooth side under tension), with both Weibull moduli close to m ≈ 20, as shown in Figure 6a. The Weibull parameters determined for both SOC0 orientations were very similar, revealing that the surface quality had a statistically negligible influence on the mechanical response of the electrolyte but still was detectable by the method used. 

For the SOC1 samples, corresponding to the electrolyte with addition of the barrier layer on the rough electrolyte side, the characteristic strength calculated was *σ*_0_ = 1956 MPa when the electrolyte smooth side was on the tensile side and *σ*_0_ = 763 MPa when the barrier was in tension. While the strength of the electrolyte side was comparable to the one obtained for the uncoated 3YSZ electrolyte, a pronounced strength decrease was obtained when the barrier layer was exposed to the tensile load. Even if the thickness of the GDC (Gadolinium Doped Ceria) barrier layer (≈10 µm) was small compared to the electrolyte thickness (≈90 µm), its effect on the resulting strength was remarkable.

The addition of the fuel electrode resulted in the approach of the strength distribution plots of the two orientations, while the GDC barrier side nearly maintained its previous strength (*σ*_0_ = 730 MPa), and the electrolyte side, now coated with the NiO/GDC cermet layer, underwent a drastic weakening (from *σ*_0_ = 1956 MPa to *σ*_0_ = 775 MPa). 

Finally, the presence of the LSCF air electrode had a minor influence on the mechanical response of the layered structure. The Weibull strengths determined were *σ*_0_ = 843 MPa and *σ*_0_ = 802 MPa when the fuel electrode and the air electrode were in tension, respectively. 

Table 4 reports the values of the measured flexural strength and Weibull modulus of each sample, for both orientations.

Comparing the stress levels in the bare electrolyte (SOC0) with the one of the whole cell (SOC3), it is clear that the strength of the electrolyte on the cell level was significantly reduced. The stress in the electrolyte at the failure of the cell was less than half that of the uncoated electrolyte. In order to understand the reasons behind the weakening phenomenon, fractographic analyses using scanning electron microscopy on selected specimens were performed.

### 3.2. Fractographic Analysis

**Figure 7:**
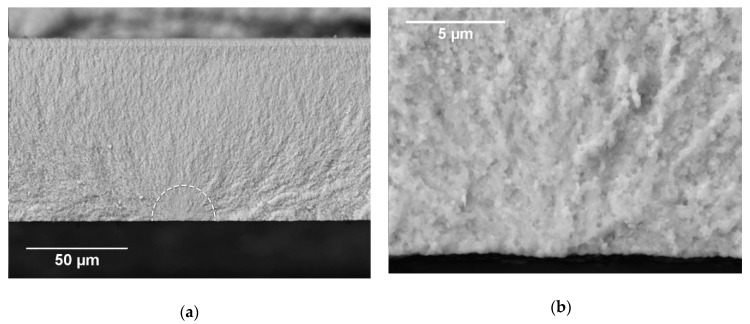
SEM images of the fracture surface of a 3YSZ specimen tested in B3B with the rough surface in tension. The dashed line in (**a**) highlights the fracture initiation site. (**b**) Magnified image of the initiation site.

The selected most significant (characteristic) fracture surfaces are reported below. The fracture surface in Figure 7 belongs to a 3YSZ specimen tested with the rough side in tension. The fracture initiation site is highlighted by the dashed line. From the high-magnification micrograph in Figure 7b, it is possible to state that the crack propagation in the vicinity of the free surface was mainly inter-granular, revealing a weak bonding strength between grains, probably caused by the manufacturing process; it then became rather trans-granular in the interior of the material. The same fracture mechanism was observed for electrolyte specimens tested with the smooth side on the tensile side of loading; this result is in agreement with the similar fracture strengths measured via the flexural test for both orientations. As expected, for all the tested single-layer 3YSZ electrolyte samples, the fracture initiated on the tensile surface of the specimen, within the area of maximum stress determined via FEM (see Figure 5).

**Figure 8:**
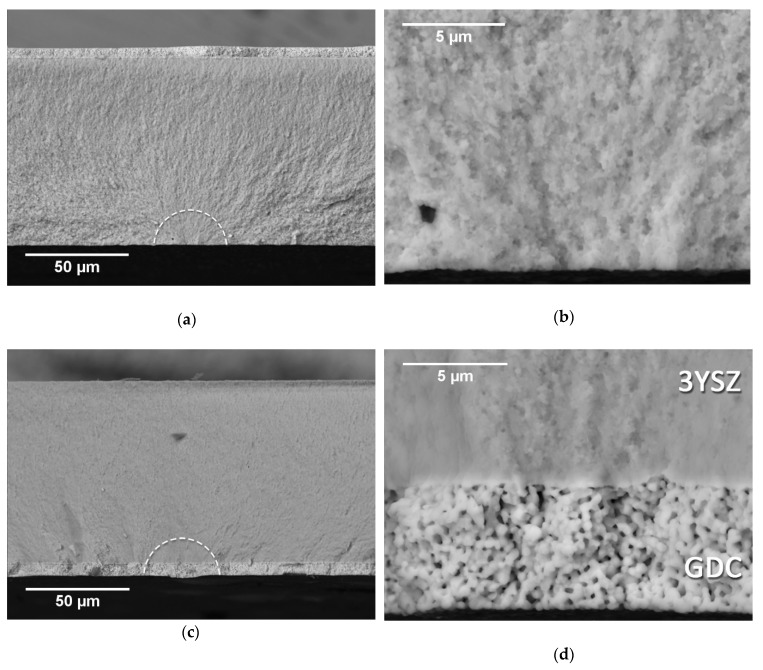
SEM images of the fracture surface of a SOC1 specimen tested in B3B with the electrolyte side in tension in (**a**) and (**b**), and with the GDC barrier layer in tension in (**c**) and (**d**). The fracture initiation areas are marked by the white dashed lines.

Figure 8 shows the fracture surface of two SOC1 specimens, consisting of the electrolyte with the addition of the GDC barrier layer on the rough surface. 

The micrographs in Figure 8a,b illustrate the fracture mechanism of this bi-layered specimen when tested with the electrolyte in tension. The fracture pattern was the same as the one observed for the single-layer electrolyte, meaning that the presence of the barrier layer on the top surface had no influence in the cracking mode. Hence, the similar characteristic strength measured during the flexural test. Figure 8c,d report the fracture surfaces belonging to SOC1 specimens, this time being tested with the GDC layer on the tensile side of the loading. The fracture seemed to initiate in correspondence with the outer surface of the barrier layer. The magnified image in Figure 8d clearly shows how the crack propagates with continuity through both the electrolyte and the barrier layer, revealing a high bonding strength between the two layers. 

**Figure 9:**
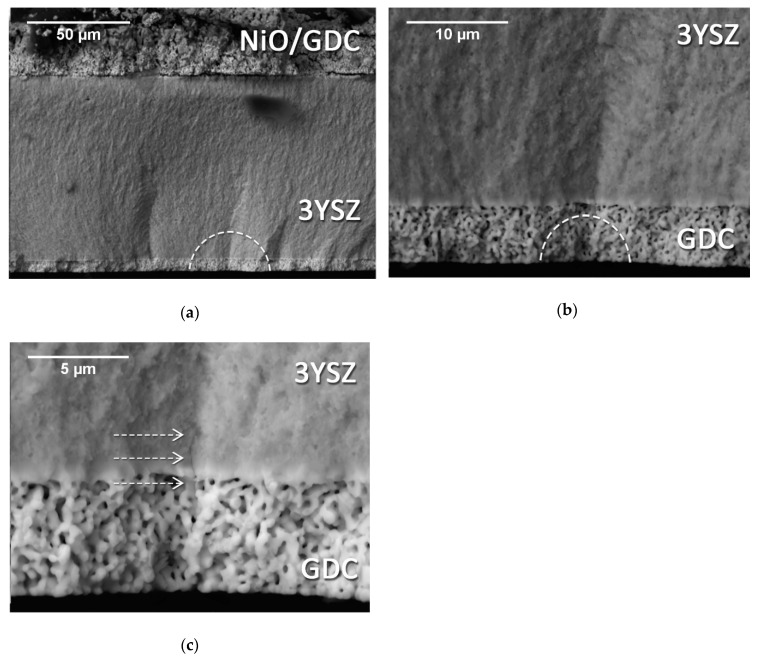
SEM micrographs of the fracture surface of a SOC2 specimen tested with the GDC barrier layer on the tensile side of loading. (**a**): Overview of the fracture surface. (**b**) Fracture initiation site located on the outer surface of the GDC layer. (**c**) The arrows indicate the “third-branch” of the crack.

Figure 9 illustrates the fracture surface of a SOC2 specimen, tested with the GDC barrier layer on the tensile side of loading. Observing Figure 9a, it is possible to notice partial delamination of the fuel electrode in the compressive area (i.e., upper part of the specimen): a continuous crack runs along the interface between the YSZ electrolyte and the NiO/GDC fuel electrode. Figure 9b shows the fracture starting site located at the outer surface of the specimen and propagating from the GDC layer into the electrolyte; this behavior reveals a good bonding between the two layers like that already observed for the SOC1 samples. It should be noted that the presence of the fuel electrode on the compressive side of loading had no visible influence on the fracture behavior; indeed, the fracture surface looks very similar to the one shown in Figure 8. This is in agreement with the experimental results, which revealed that SOC1 and SOC2 samples had, to a good approximation, the same flexural strength when tested with the GDC layer on the tensile side of loading was. The specimen under investigation broke in two pieces, but a clear “third-branch” of the crack propagating through the electrolyte is present and highlighted by the arrows in Figure 9c. 

**Figure 10:**
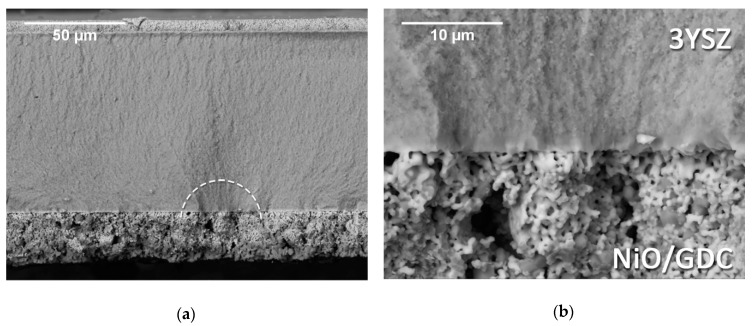
(**a**) Example of the fracture surface appearance of a SOC2 specimen tested with the fuel electrode on the tensile side of loading; fracture. (**b**) Detail of the fracture initiation area.

An example of the fracture surface of a SOC2 specimen tested with the fuel electrode in tension is illustrated in Figure 10. This time, no delamination was observed. The fracture initiation site seemed to be located somewhere between the fuel electrode and the electrolyte; however, the porous nature of the electrode layer makes the exact identification of the initiation point impossible. As shown in Figure 10b, the crack propagated with continuity from the fuel electrode into the electrolyte, meaning that the bonding between these layers was strong enough and the crack did not deflect along the interface. This could explain the strength decrease measured for the SOC2 samples when tested with the fuel electrode in tension. Considering that the fuel electrode was weaker than the electrolyte, it would start cracking at lower applied stress; since these cracks were not able to deflect along the interface with the electrolyte because of the strong bonding, they would penetrate into the electrolyte. This mechanism seemed to have a detrimental effect on the strength of the layered structure analyzed. Similar fracture mechanism for SOC3 specimens tested on the same orientation (i.e. with the fuel electrode in tension) was observed. 

In Figure 10a, the effect of compressive stresses acting on the upper part of the specimen can be observed at the interface between the electrolyte and the barrier layer. 

**Figure 11:**
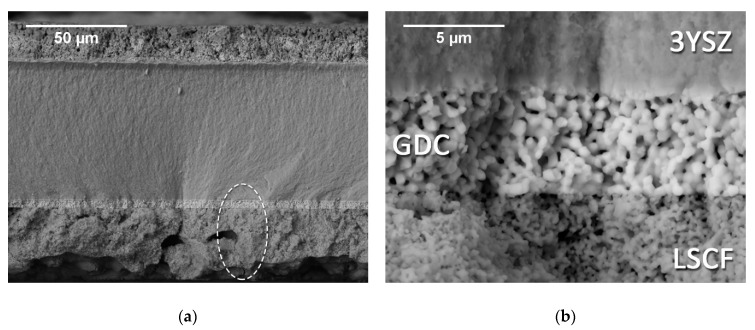
SEM images showing a typical fracture surface of a whole MEA (SO3) tested with the air electrode side in tension. The circle in (**a**) highlights a pre-crack in the air electrode. (**b**) Detail of the fracture initiation site at the interface between the LSCF air electrode and the GDC barrier layer.

The micrographs in Figure 11 illustrate a typical fracture surface of the whole MEA (SOC3), tested with the air electrode on the tensile side of loading. The fracture initiation site was most likely located at the interface between the LSCF (Lanthanum Strontium Cobalt Ferrite) air electrode and the GDC barrier layer, in correspondence with the area of maximum stress calculated via FEM. A detail of this area is shown in Figure 11b. Before the failure, some cracks formed in the air electrode layer and they expanded up to the substrate interface. The pre-cracking of the air electrode was a consequence of the much lower strength of this layer in comparison to the electrolyte. Because of residual stresses derived from the mismatch of the thermal expansion coefficients, the air electrode encountered itself already in tension before the mechanical test; given that this layer was really porous and mechanically weak, it was not expected to support a much higher tensile stress during the flexural test. Therefore, it was likely to pre-crack at low applied stress levels. The dashed circle ellipse in Figure 11a highlights an example of a pre-crack starting at the surface and propagating to the interface with the barrier layer being out of the failure initiation site. Such cracks only formed locally, therefore their influence on the overall stiffness was negligible and the force–displacement curve would not show any evident deflection from linearity. However, they would act as stress concentrators during external loading and might lead to an early failure of the laminate, when in correspondence with defects in the substrate. As a result, the failure stress and the characteristic strength of the electrolyte on the cell level were much lower (more than twice) than those of the uncoated electrolyte samples, which is in good agreement with the literature [7,10]. However, the whole cell showed a strength increase in comparison to the strength values measured for SOC2 specimens.

## 4. Discussion

### 4.1. Effect of Residual Stresses

The cell was made up of co-sintered functional self-supported layers. As emerged from the fractographic analyses, the interfaces generated between adjacent layers might influence the fracture mechanism of the cell. However, layer interfaces were not the only factor responsible for the changes in the mechanical resistance; a significant role was ascribed to residual stresses developed during processing. Because of the CTE (Coefficient of Thermal Expansion) mismatch, residual stresses arose in the layers during cooling from the sintering temperature as the layers were bonded to each other and were not allowed to shrink freely. The main difference with layered systems studied in the literature where co-sintering of green bodies were usually investigated [30,31,32], contrary to the cell case where already sintered electrolyte is subjected to co-sintering with green bodies of added layers. Therefore, the multi-layered samples were not in a stress-free state at room temperature before mechanical loading during B3B tests. Some of the layers were already in tension while others were in compression. These residual stresses were responsible for a more fragile cell when handling it [33]. For the proper evaluation of the room-temperature strength of the materials under investigation, such residual stresses should be taken into account. The effect of residual stresses on the strength of bi-layer SOC materials has already been investigated; it has been reported that residual stresses could either strengthen or weaken the layered structure, depending on which of the layers is exposed to tensile loading. In fact, the residual stresses present in the layers redistribute the stress field created by an external load applied. However, literature data deals only with bi-layer or symmetrical structures; the influence of residual stresses on the mechanical integrity of the whole cell has not been reported yet. 

Looking at the results reported in Table 4 for SOC1 samples, it can be observed that the strength of the bi-layered structure slightly increased when the electrolyte was on the tensile side of loading, but it drastically decreased when the electrolyte was on the compressive side. The reason for this behavior can be found in the residual stresses that developed in the sample after cooling down from the sintering temperature. A finite element model allowed for the estimation of the stresses inside the layers: at room temperature, the electrolyte was in a compressive state (about 50 MPa), while the GDC barrier layer was solicited by high tensile stresses of about 600 MPa. Therefore, the barrier layer was already in tension before the flexural test; hence, the applied stress necessary to reach failure was lower. On the contrary, the compressive stresses developed in the electrolyte acted against the tensile stresses applied during mechanical testing, resulting in a strength increase. The high residual stresses derived from the CTE mismatch in the non-symmetrical laminate would be partially released by elastic deformation of the whole laminate, resulting in non-planar (curved) samples. Moreover, the different strength of individual materials should be taken in to account depending on the fracture initiation place. Indeed, fracture might not occur in the vicinity of the tensile outer surface as is usual in case of monolithic materials tested (see SOC0 as an example). 

The addition of the fuel electrode resulted again in nominal residual compressive stresses generated in the electrolyte (about 95 MPa) and tensile stresses in both the outer layers (465 MPa in the barrier layer and 90 MPa in the fuel electrode). Despite the addition of the new layer, a negligible difference between the mechanical response of SOC1 and SOC2 samples tested with the barrier layer on the tensile side of loading was observed. This might be a consequence of three aspects: first, the delamination of the fuel electrode when on the tensile side of loading (see Figure 9); second, the residual stresses acting in the electrolyte and barrier layer, which were of the same magnitude of those acting in SOC1 samples; finally, the significantly higher compliance of the electrode layer. 

In contrast, when SOC2 samples were tested in the opposite orientation, the presence of the fuel electrode in a pre-tensed state played an important role in the strength of the tri-layered structure: the Weibull characteristic strength calculated for SOC2 samples became significantly smaller than the one determined for SOC1 samples. This strength loss was caused by the electrode porosity, which allowed cracking at lower applied stresses due to its weak nature (lower strength and stress concentration effect on pores) and the residual tensile stresses, which were independent of the porosity. Such cracks propagated across the thickness until reaching the electrolyte without fracture of the whole layered system; there they encountered a strong and brittle interface generated during the sintering process (see Figure 10). Thus, pre-cracks were not able to deflect along the interface and act as stress concentrators finally propagating into the electrolyte, with a consequent loss of mechanical strength. 

With the addition of the air electrode, the stress distribution within the layers was analogous to the one described for SOC2 samples: the electrolyte was in compression, while all the other layers around it were in tension. In particular, the air electrode was pre-stressed with a tensile stress of about 180 MPa; given that its room-temperature strength was approximately 160 MPa [34,35], this would explain the cracks observed in Figure 11.

With respect to the final mechanical strength, the presence of the LSCF air electrode played a minor role. Indeed, the characteristic strength measured for SOC3 samples was slightly higher, but still of the same order of magnitude of the one obtained for the SOC2 samples. This is in accordance with the fuel electrode having low residual stresses, low elastic modulus, and low strength [10]. The increase was a consequence of the stress redistribution within the functional layers. This means that the pre-cracks forming in the air electrode layer for relatively low applied stresses were not able to penetrate into the electrolyte and they had no significant influence on the failure mechanism.

### 4.2. Stresses across the Thickness

**Figure 12:**
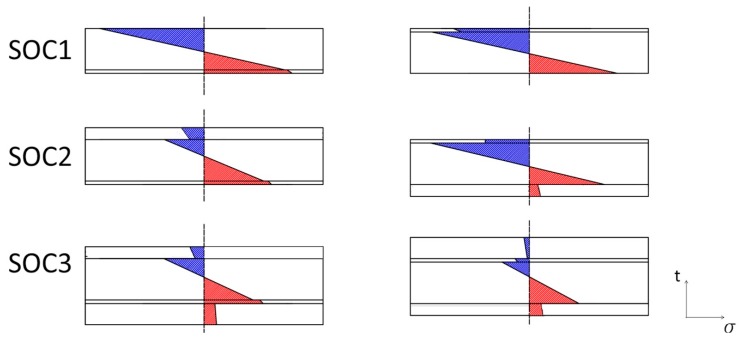
Stress distribution across the section of the layered samples tested in biaxial flexure. Compressive stresses are indicated in blue, tensile stresses in red.

All the analyzed samples fractured within the area of maximum applied stress indicated in Figure 5. For a better identification and explanation of the fracture initiation site across the section of the samples, an FEM was used. In particular, the FEM of the biaxial flexural test allowed the estimation of the stress distribution across the section corresponding to a certain applied load. Figure 12 shows how the stresses distribute across the section of every sample in a fully elastic regime with low surface curvature. In these results, an estimation of residual stresses developed during processing was taken into account. They were incorporated as a separate step in the model before calculating the biaxial flexural stresses. The cross-sectional distribution of the total stresses revealed that the maximum tensile stress was not always located at the surface under a tensile load. Indeed, in some cases, the maximum tensile stress arose at the interface between layers. This means that the fracture would not always initiate at the external surface, but it could start at the most stressed interface between layers. This was particularly evident for the SOC3 sample oriented with the air electrode on the tensile side of loading. In this case, the maximum tensile stress was located at the interface between the LSCF electrode and the GDC barrier layer and the tensile stresses in the air electrode were pretty small compared to those developed in the barrier layer. Therefore, if the tensile stress present in the air electrode did not exceed the material strength, the fracture would initiate at the interface between electrode and barrier layer. The FEM results were used to confirm and to explain the observed fracture behavior using fractographic analysis discussed previously in this work.

## 5. Conclusions

In this work, electrolyte-supported SOC cells that are currently used in commercial stacks have been investigated with respect to their biaxial flexural strength at room temperature. It can be concluded that the strength of the supportive electrolyte on the cell level is sensibly reduced. The reason for the mechanical loss is a consequence of two main phenomena:

- The formation of strong interfaces and constraints between adjacent functional layers during manufacturing, and especially during the sintering process. Such interfaces, due to their high fracture energy, will impede the deflection of the crack formed in the porous electrodes to deflect at the interface with the electrolyte. The tip of such cracks may act as stress concentrators at the electrolyte interface and they might easily penetrate into the electrolyte, thus lowering the final strength.
- Residual stresses arising in the different layers of the cell as a consequence of the thermal expansion mismatch. Such stresses will redistribute with the addition of layers to the electrolyte and will act against or in favor of the externally applied load affecting the resulting strength.

## Figures and Tables

**Table 1:** List of the cell layers with their composition and nominal thickness.

Layer	Material	Composition	Thickness (µm)
Electrolyte	3YSZ	(Y_2_O_3_)_0.03_(ZrO_2_)_0.97_	90
Barrier layer	20GDC	Gd_0.2_Ce_0.8_O_2_	10
Fuel Electrode	NiO/10GDC	(NiO)/(Gd_0.1_Ce_0.9_O_2_)	27
Air Electrode	LSCF	La_0.6_Sr_0.4_Co_0.2_Fe_0.8_O_3-δ_	45

**Table 2:** List of the layered structures characterized with their given names, brief description, and nominal thicknesses.

Sample	Name	Description	t (µm)
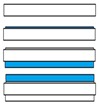	SOC0	Electrolyte	90
	SOC1	Electrolyte + Barrier	100
	SOC2	Electrolyte + Barrier + Fuel Electrode	127
	SOC3	Electrolyte + Barrier + Fuel Electrode + Air Electrode	172

**Table 3:** List of the cell layers with their composition and nominal thickness.

Layer	Material	E (GPa)	ν (-)	ρ (g/cm^3^)	α (K^−1^)
Electrolyte	3YSZ	202.5	0.27	6.05	10.8 × 10^−6^
Barrier Layer	20GDC	120	0.26	4.02	12.5 × 10^−6^
Fuel Electrode	NiO/10GDC	120	0.25	5.97	13.4 × 10^−6^
Air Electrode	LSCF	80	0.30	2.36	16.6 × 10^−6^

**Table 4:** Compilation of Weibull parameters obtained from the biaxial bending test, including the 95% confidence intervals.

Sample	Tested Surface	σ_0_ (MPa)	m
SOC0	Smooth	1854.4 (1818.8|1889.7)	19.5 (14.7|25.5)
	Rough	1818.9 (1782.2|1855.3)	18.0 (13.7|23.4)
SOC1	Electrolyte	1955.9 (1901.2|2010.7)	12.2 (9.4|15.7)
	Barrier	762.6 (747.2|777.9)	16.8 (13.0|21.5)
SOC2	Fuel Electrode	775.0 (762.4|783.3)	24.5 (19.0|31.1)
	Barrier	729.5 (715.4|743.5)	17.4 (13.5|22.1)
SOC3	Fuel Electrode	844.4 (838.9|849.7)	57.4 (43.4|74.8)
	Air Electrode	801.7 (790.9|812.4)	25.9 (19.9|33.3)
